# Association between Postoperatively Developed Atrial Fibrillation and Long-Term Mortality after Esophagectomy in Esophageal Cancer Patients: An Observational Study

**DOI:** 10.1371/journal.pone.0154931

**Published:** 2016-05-05

**Authors:** Ji-Hyun Chin, Young-Jin Moon, Jun-Young Jo, Yun A. Han, Hyeong Ryul Kim, Eun-Ho Lee, In-Cheol Choi

**Affiliations:** 1 Department of Anaesthesiology and Pain Medicine, Asan Medical Center, University of Ulsan College of Medicine, Seoul, Korea; 2 Department of Thoracic and Cardiovascular Surgery, Asan Medical Center, University of Ulsan College of Medicine, Seoul, Korea; Baylor College of Medicine, UNITED STATES

## Abstract

**Background:**

Newly developed atrial fibrillation (AF) in patients who have undergone an esophagectomy increases the incidence of postoperative complications. However, the clinical implications of AF have not been fully elucidated in these patients. This retrospective observational study investigated the predictors for AF and the effect of AF on the mortality in esophageal cancer patients undergoing esophagectomy.

**Methods:**

This study evaluated 583 patients undergoing esophagectomy, from January 2005 to April 2012. AF was defined as newly developed postoperative AF requiring treatment. The risk factors for AF and the association between AF and mortality were evaluated. The long-term mortality was the all-cause mortality, for which the cutoff date was May 31, 2014.

**Results:**

AF developed in 63 patients (10.8%). Advanced age (odds ratio [OR] 1.099, 95% confidence interval [CI] 1.056–1.144, *P* < 0.001), preoperative calcium channel blocker (CCB) (OR 2.339, 95% CI 1.143–4.786, *P* = 0.020), and angiotensin-converting enzyme inhibitor (ACEI) or angiotensin receptor blocker (ARB) (OR 0.206, 95% CI 0.067–0.635, *P* = 0.006) were associated with the incidence of AF. The Kaplan-Meier curve showed a significantly lower survival rate in the AF group compared to the non-AF group (*P* = 0.045), during a median follow-up of 50.7 months. The multivariable analysis revealed associations between AF and the 1-year mortality (hazard ratio [HR] 2.556, 95% CI 1.430–4.570, *P* = 0.002) and between AF and the long-term mortality (HR 1.507, 95% CI 1.003–2.266, *P* = 0.049).

**Conclusions:**

In esophageal cancer patients, the advanced age and the preoperative medications (CCB, ACEI or ARB) were associated with the incidence of AF. Furthermore, postoperatively developed AF was associated with mortality in esophageal cancer patients after esophagectomy, suggesting that a close surveillance might be required in patients who showed AF during postoperative period.

## Introduction

Esophageal cancer is the eighth most common cancer worldwide, demonstrating a poor prognosis [1]. The mainstay treatment for this type of cancer is esophagectomy [2,3]. However, there is a still considerable risks of morbidity and mortality after an esophagectomy [4,5], although postoperative care has improved. Atrial fibrillation (AF) is a postoperative morbidity with an incidence of 9–46% [6–11]. One concern about AF after esophagectomy is its association with other postoperative complications. The clinical characteristics and prognosis in AF patients have been investigated in lung cancer patients undergoing pulmonary lobectomy, suggesting the detrimental effects of AF on morbidity and mortality [12]. However, the effects of AF following esophagectomy have not been fully evaluated, despite a substantially unfavourable burden of AF. This might be due to a lack of data from studies that exclusively enrolled patients undergoing esophageal cancer surgery, without other non-cardiac thoracic surgeries. It would be useful to identify the clinical significances of AF after esophagectomy in esophageal cancer patients, when considering the different prognoses between various types of cancers.

There are few studies that have evaluated the association between AF and the postoperative complications, such as anastomotic leakage and pulmonary complications, in patients who had undergone esophagectomy [10,13]. There was also a lack of studies on addressing the effects of AF on the long-term mortality in patients who have undergone esophagectomy [14].

We aimed to investigate whether postoperatively developed AF was associated with mortality in a large number of patients who underwent esophagectomy due to esophageal cancer. In addition, we evaluated the perioperative risk factors for AF after esophagectomy in these patients.

## Materials and Methods

This retrospective cohort study examined 598 consecutive patients who underwent esophagectomy due to esophageal cancer at Asan Medical Center, Seoul, South Korea, from January 2005 to April 2012. Patients with AF, atrial flutter, paced rhythm on preoperative electrocardiogram, and history of AF were excluded. The research protocol was approved and the requirement of written informed consent was waived by our Institutional Review Board (AMC IRB 2013–0959).

The patient data were obtained through the review of electronic medical records. The clinical data included age, gender, body mass index (BMI) (kg/m^2^), preoperative electrocardiogram, preoperative resting heart rate (HR), diabetes mellitus (DM), hypertension, ischemic heart disease (IHD), cerebrovascular disease, peripheral vascular disease (PVD), chronic kidney disease (serum creatinine > 1.5 mg/dl or estimated glomerular filtration rate < 60 ml/min/1.73 m^2^), liver disease, history of neoadjuvant chemoradiation therapy, classification of the American Society of Anesthesiologist physical status, preoperative hematocrit, left ventricular ejection fraction, predicted forced vital capacity (FVC), predicted forced expiratory volume in one second (FEV_1_), FEV_1_/FVC ratio, duration of surgery, and infused fluid volume during surgery. The data regarding the preoperative use of angiotensin-converting enzyme inhibitor (ACEI) or angiotensin receptor blocker (ARB), *β*-blocker, calcium channel blocker (CCB), insulin, oral hypoglycemic agents, lipid-lowering drugs, aspirin, and diuretics were acquired. The pathologic stage of the esophageal cancer was determined using the TNM classification of the 7^th^ edition of the American Joint Committee on Cancer. The type of surgical approach was divided into two-stage, three-stage, and transhiatal esophagectomy. The data regarding the postoperative outcomes included pneumonia (diagnosed with radiographic evidence of pneumonia) [14], acute kidney injury, hospital stay, in-hospital mortality (mortality during hospital stay or within 30 days after surgery), 1-year mortality, and long-term mortality. Acute kidney injury (AKI) was classified according to the Acute Kidney Injury Network (AKIN) criteria for changes in the level of serum creatinine within 48 hours of surgery [15]. The long-term mortality was the all-cause mortality, for which the cutoff date was May 31, 2014.

AF was defined as newly developed AF after esophagectomy prior to discharge that required therapy irrespective of the AF duration. AF was reviewed during the postoperative period, or from the patient arrival at the intensive care unit after surgery until discharge. The rhythm was monitored continuously with lead II and V5 electrocardiogram in the intensive care unit. In the general ward, the electrocardiogram was checked once a day routinely and when the patients experienced newly developed symptoms, such as palpitation or dizziness, or when the physical examination disclosed an irregular rhythm. Electrical cardioversion or amiodarone administration (300 mg of intravenous bolus administration followed by 1500 mg/day for 24 hours) was performed to restore the sinus rhythm.

### Statistical Analysis

The continuous data were expressed as mean ± standard deviation for the normally distributed data or median (interquartile range) for the nonparametric data, and the categorical data as frequencies (percentages). The continuous variables were compared by using the *t*-test or Mann-Whitney *U* test for parametric and nonparametric variables, respectively. The categorical variables were compared by using the chi-square test or the Fisher's exact test, as appropriate.

The crude and adjusted risks for AF were compared by using univariate and multivariable logistic regression analyses, and odds ratios (ORs) with 95% confidence intervals (CIs) were calculated. The variables with a P value ≤ 0.10 in the univariate analysis and those that were likely to have an association with AF were included in the multivariable logistic regression model with backward elimination. Because preoperative HR and IHD are known risk factors for the postoperative AF, these were forced in to the model. Three multivariable logistic regression models with backward elimination process were constructed, as follows; model 1 included age, HR, IHD, ACEI or ARB and CCB; model 2 included age, IHD, ACEI or ARB, β-blocker and CCB; model 3 included age, HR, IHD, ACEI or ARB, β-blocker and CCB. The selected variables for the final model were age, HR, ACEI or ARB, and CCB. Model discrimination was evaluated by using C statistic and model calibration was evaluated by using Hosmer-Lemeshow goodness-of-fit test. The final model showed C statistics = 0.722 and *P* = 0.227 of Hosmer-Lemeshow goodness-of-fit test.

Long-term survival was evaluated by using Kaplan-Meier estimates and compared with the log-rank test. Crude risk factors for mortality were compared with univariate Cox proportional hazards regression analysis by using the covariates listed in Tables 1 and 2. To evaluate the independent effects of AF on mortality, a multivariable Cox proportional hazards regression model with backward elimination was constructed with related survival time as a function of AF, adjusting variables with *P* values ≤ 0.10 in the univariate analyses, and the adjusted hazard ratios (HRs) with 95% CIs were calculated. The preoperative BMI, hematocrit, pathologic stage of cancer, postoperative pneumonia, and AKI were adjusted in the multivariable Cox proportional hazards model to evaluate the effect of AF on the 1-year mortality. The preoperative BMI, DM, hematocrit, history of PVD, pathologic stage of cancer, postoperative pneumonia, and AKI were adjusted in the multivariable Cox proportional hazards model to evaluate the effect of AF on the long-term mortality. Because postoperative sepsis was likely to include multiple major organ dysfunctions such as lung and kidney, it was not included in the multivariable model although it showed a statistical significance in the univariate analysis. In addition, we analyzed the data after excluding the in-hospital mortality to eliminate the effect of AF on the early perioperative mortality. The preoperative BMI, DM, hematocrit, and pathologic stage of cancer were adjusted in the multivariable Cox proportional hazards model to evaluate the effect of AF on the 1-year mortality excluding the in-hospital mortality. The proportion hazards assumption was confirmed by examining log (-log[survival]) curves and by testing the Schoenfeld residuals, and no relevant violations were found.

**Table 1:** Baseline Demographic and Clinical Characteristics.

	AF (n = 63)	No AF (n = 520)	*P*
Age (years)	67.0 (63.0–71.0)	62.0 (56.0–68.0)	< 0.001
Female sex	1 (1.6)	34 (6.5)	0.200
Body mass index (kg/m^2^)	22.6 ± 3.0	23.1 ± 3.0	0.149
Medical history			
Diabetes mellitus	14 (22.2)	85 (16.3)	0.319
Hypertension	23 (36.5)	164 (31.5)	0.425
Ischemic heart disease	2 (3.2)	19 (3.7)	0.847
Cerebrovascular disease	2 (3.2)	15 (2.9)	0.789
Peripheral vascular disease	2 (3.2)	21 (4.0)	0.992
Chronic kidney disease	0 (0)	21 (4.0)	0.205
Liver disease	7 (11.1)	76 (14.6)	0.575
Chemo-radiation therapy	32 (50.8)	210 (40.4)	0.148
ASA PS			0.637
1	3 (4.8)	34 (6.5)	
2	58 (92.0)	477 (91.7)	
3	2 (3.2)	9 (1.7)	
Preoperative data			
Resting heart rate (beats/min)	75.0 (66.0–85.8)	73.5 (66.0–81.0)	0.275
Hematocrit (%)	38.3 (34.2–40.4)	38.6 (35.2–41.6)	0.254
LVEF (%)	61.0 (58.0–65.3)	62.0 (59.0–65.0)	0.480
Predicted FVC (%)	92.0 (83.8–100.3)	93.0 (85.5–102.0)	0.533
Predicted FEV_1_ (%)	93.0 (83.0–103.0)	94.0 (84.0–104.0)	0.563
FEV_1_/ FVC ratio (%)	71.0 (65.0–76.0)	75.0 (69.0–80.0)	0.005
Preoperative medications			
ACEI or ARB	4 (6.3)	78 (15.0)	0.094
β-blocker	7 (11.1)	29 (5.6)	0.148
CCB	14 (22.2)	71 (13.7)	0.103
Dihydropyridine type CCB	10 (15.9)	61 (11.8)	0.456
Insulin	14 (22.2)	80 (15.4)	0.225
Oral hypoglycemic agent	7 (11.1)	46 (8.8)	0.720
Lipid-lowering agent	4 (6.3)	45 (8.7)	0.702
Aspirin	1 (1.6)	9 (1.7)	0.667
Diuretics	4 (6.3)	43 (8.3)	0.777
Intraoperative characteristics			
Anesthesia time (minutes)	465.0 (390.0–520.0)	450.0 (365.0–517.0)	0.283
Crystalloid (ml)	1600.0 (1050.0–2000.0)	1350.0 (1000.0–2000.0)	0.309
Colloid (ml)	1000.0 (1000.0–1400.0)	1000.0 (900.0–1300.0)	0.332
Surgical procedures			0.517
Two-stage	42 (66.7)	372 (71.5)	
Three-stage	20 (31.7)	145 (27.9)	
Transhiatal	1 (1.6)	3 (0.6)	
Minimally invasive surgery	5 (7.9)	61 (11.7)	0.369
Pathologic stage of cancer			0.235
T0	18 (28.6)	93 (17.9)	
Stage 0, I, and II	35 (55.6)	338 (65.0)	
Stage III and IV	9 (14.2)	82 (15.8)	
Unknown	1 (1.6)	7 (1.3)	

Values are mean ± standard deviation, median (interquartile range), or number (percentage). AF = atrial fibrillation; ASA PS = American Society of Anesthesiologist physical status; LVEF = left ventricular ejection fraction; FVC = forced vital capacity; FEV_1_ = forced expiratory volume in one second; ACEI = angiotensin converting enzyme inhibitor; ARB = angiotensin receptor blocker; CCB = calcium channel blocker.

**Table 2:** Outcomes after Esophagectomy in Esophageal Cancer Patients.

	AF (n = 63)	No AF (n = 520)	*P*
Pneumonia	22 (34.9)	71 (13.7)	< 0.001
AKI (AKIN criteria)			0.492
No AKI	40 (63.5)	340 (65.4)	
Stage Ⅰ	19 (30.2)	155 (29.8)	
Stage Ⅱ	1 (1.6)	15 (2.9)	
Stage Ⅲ	3 (4.8)	10 (1.9)	
Anastomotic leakage	5 (7.9)	28 (5.4)	0.408
Sepsis	22 (34.9)	79 (15.2)	< 0.001
Hospital stay (days)	15.0 (12.0–23.0)	12.0 (11.0–17.0)	< 0.001
In-hospital mortality	7 (11.1)	11 (0.2)	< 0.001

Values are median (interquartile range), or number (percentage). AF = atrial fibrillation; AKI = acute kidney injury; AKIN = acute kidney injury network.

A *P* value < 0.05 was considered statistically significant. All the statistical analysis was performed using SAS software version 9.1 (SAS Institute, Cary, NC).

## Results

Of the 598 patients, 15 were excluded: 4 who underwent pharyngeal resection in combination with esophagectomy, 5 who underwent exploratory thoracotomy, and 6 who had preoperative AF or atrial flutter. The remaining 583 patients (555 with squamous cell carcinoma, 21 with adenocarcinoma, and 7 with other histological types of esophageal cancer; 3 with melanoma, 1 with signet ring cell carcinoma, 1 with lymphoepithelioma-like carcinoma, 1 with sarcomatoid carcinoma, and 1 with small cell carcinoma) were evaluated. The patient’s demographics and perioperative variables are shown in Table 1.

**Fig 1:**
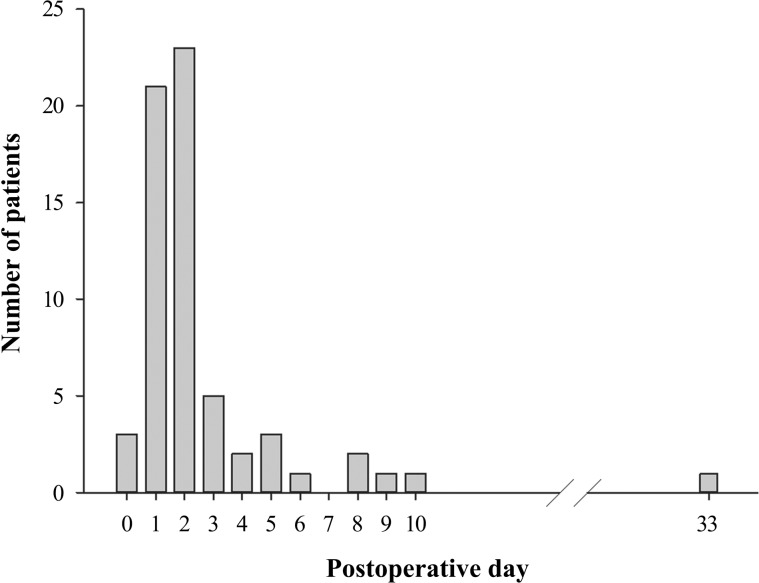
The Number of Patients who Developed Atrial Fibrillation after Esophagectomy.

AF developed in 63 patients (10.8%). AF occurred within 3 days after esophagectomy in 82.5% of AF patients, and the AF incidence peaked at postoperative day 2 (Fig 1).

The patients who suffered from AF were older and had a lower FEV_1_/ FVC ratio compared to those who did not (Table 1). In addition, the patients who showed AF showed a longer hospital stay and higher incidences of postoperative pneumonia, sepsis, and in-hospital mortality compared to those who did not (Table 2). Specifically, there was an association between AF and pneumonia (unadjusted OR 3.393, 95% CI 1.909–6.032, *P* < 0.001).

### Perioperative Risk Factors for AF after Esophagectomy

The multivariable logistic regression analysis determined that the advanced age (OR 1.099, 95% CI 1.056–1.144, *P* < 0.001), and the preoperative CCB medication (OR 2.339, 95% CI 1.143–4.786, *P* = 0.020) were independent risk factors for AF, and that the preoperative ACEI or ARB medication (OR 0.206, 95% CI 0.067–0.635, *P* = 0.006) had a beneficial effect on AF development.

### Effect of AF on Mortality and Other Risk Factors for Mortality

**Fig 2:**
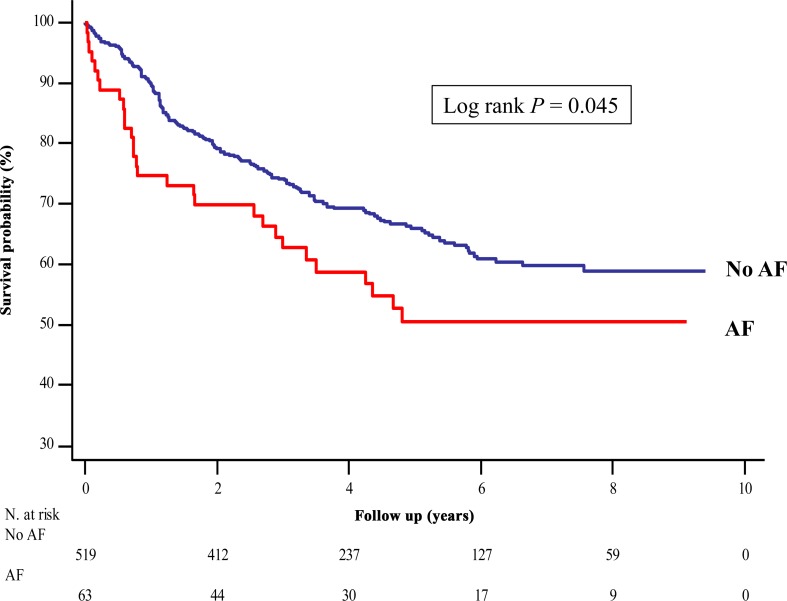
Kaplan-Meier Survival Curve after Esophagectomy in the Esophageal Cancer Patients. The survival rate in the AF group was significantly lower compared to that in the non-AF group (log rank *P* = 0.045). AF = atrial fibrillation.

Twenty-nine and 176 patients died in the AF and non-AF group, respectively, during a median follow-up of 50.7 months (interquartile range: 31.6–78.3 months). The Kaplan-Meier curve demonstrated that 1-, 3-, and 5-year overall survival of AF group was 74.6%, 64.5%, and 50.5%, and, 1-, 3-, and 5-year overall survival of non-AF group was 89.8%, 74.1%, and 65.9%, respectively, identifying a significantly lower survival rate in the AF group compared to the non-AF group (*P* = 0.045) (Fig 2).

The 1-year mortality rate was 25.4% (n = 16) in the AF group and 10.2% (n = 52) in the non-AF group. The Cox proportional hazard model analysis demonstrated that there was an association between AF and the 1-year mortality (unadjusted HR 2.773, 95% CI 1.558–4.851, *P* < 0.001; adjusted HR 2.556, 95% CI 1.430–4.570, *P* = 0.002).

Similarly, the Cox proportional hazard model analysis demonstrated that there was an association between AF and the long-term mortality (unadjusted HR 1.490, 95% CI 1.006–2.207, *P* = 0.047; adjusted HR 1.507, 95% CI 1.003–2.266, *P* = 0.049) (Table 3).

**Table 3:** Multivariable Cox Proportional Hazards Analysis of the Factors Predicting Long-term Mortality after Esophagectomy in Esophageal Cancer Patients.

	Hazard ratio	95% CI	*P*
Body mass index	0.920	0.874–0.969	0.002
Diabetes mellitus	1.621	1.152–2.279	0.006
Hematocrit	0.926	0.897–0.956	< 0.001
Peripheral vascular disease	2.419	1.422–4.113	0.001
Pathologic stage of cancer	1.719^a^	1.139–2.594	0.010
	4.175^b^	2.618–6.656	< 0.001
Postoperative AF	1.507	1.003–2.266	0.049
Postoperative pneumonia	1.990	1.432–2.765	< 0.001
Postoperative AKI (AKIN criteria)	7.107^c^	3.618–13.962	< 0.001

AF = atrial fibrillation; AKI = acute kidney injury; AKIN = acute kidney injury network; CI = confidence interval.

^a^(stage 0, I, and II) vs. T0.

^b^(stage III and IV) vs. stage 0.

^c^AKIN stage III vs. (no AKI, AKIN stage I, and AKIN stage II)

**Fig 3:**
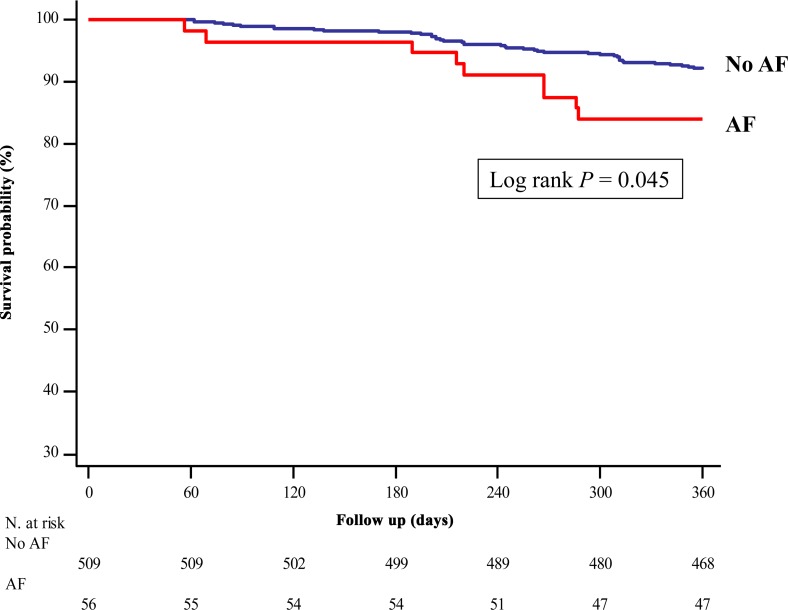
Kaplan-Meier Survival Curve within One Year after Esophagectomy in Esophageal Cancer Patients after Excluding In-hospital Death. The survival rate in the AF group was significantly lower compared to that in the non-AF group within one year following esophagectomy, after excluding the in-hospital death (log rank *P* = 0.045). AF = atrial fibrillation.

To exclude the effect of AF on the in-hospital mortality, we additionally analyzed the data after excluding the in-hospital mortality from the analysis. The Kaplan-Meier curve indicated a significantly lower survival rate in the AF group compared to the non-AF group, during 1 year follow-up, after excluding the in-hospital mortality (*P* = 0.045) (Fig 3). The Cox proportional hazard model analysis identified an association between AF and the 1-year mortality excluding the in-hospital mortality (unadjusted HR 2.056, 95% CI 1.001–4.225, *P* = 0.050; adjusted HR 2.130, 95% CI 1.026–4.422, *P* = 0.043). However, there was no association between AF and the long-term mortality after excluding the in-hospital mortality (unadjusted HR 1.218, 95% CI 0.780–1.901, *P* = 0.385).

We completed the data regarding the causes of death in 73.2% (n = 150) of the patients who died during a total follow-up period. One-hundred forty-five patients (96.6%) died of cancer-related problems (cancer progression and cancer-related or esophagectomy-related complications including pneumonia and sepsis), 1 (0.7%) of sepsis after emergent surgery to resolve iliac artery occlusion, 1 (0.7%) of pulmonary embolism, 1 (0.7%) of intracranial haemorrhage due to disseminated intravascular coagulation, 1 (0.7%) of postoperative fulminant hepatitis, and 1 (0.7%) of trauma.

## Discussion

Our present study found that newly developed AF during the postoperative period was associated with 1-year and long-term mortality in esophageal cancer patients who have undergone esophagectomy. This association is still significant regarding the 1-year mortality after excluding the in-hospital mortality. In addition, advanced age and preoperative CCB medication were associated with an increased incidence of AF, whereas preoperative ACEI or ARB medication was associated with a decreased incidence of AF.

Few studies have focused on the association between mortality and AF after esophagectomy [13,14]. The 60-day survival rate was reported to be lower in patients with AF after esophagectomy compared to the non-AF patients [13]. Another study found no significant difference in mortality after esophagectomy between the AF and the non-AF groups with a median follow-up of 39 months [14]. Our current study suggests that AF might be independently associated with mortality in esophageal cancer patients undergoing esophagectomy. The different findings of our study form the previous one might be, at least in part, attributable to the differences in the demographic characteristics, including a lower body mass index, the extreme male predominance, and a different histologic distribution. Our cohort had a very high incidence (95.3%) of squamous cell carcinoma, which is a totally different distribution from that reported in the USA and Europe [1]. Although the underlying mechanism for the association between AF and mortality has not been clearly understood, it can be speculated, as follows. First, hypoperfusion caused by AF might increase the risk for infection, such as pneumonia, and anastomosis failure, resulting in sepsis and perioperative death. Hypoperfusion also might induce occult remnant cancer, if any, to turn to an aggressive tumor [16,17], leading to a low overall survival rate during the follow-up period. Second, the patients with AF might have relatively high baseline inflammation levels which are further increased by the surgical procedure, esophagectomy, and this overwhelmed inflammation might underlie the association between AF and mortality in esophageal cancer patients [18,19]. In fact, inflammation is related to cancer development and progression [20,21]. In addition, it has been reported that AF in cancer patients is associated with cancer occurrence and cancer metastasis, suggesting the occurrence of AF as a marker of occult cancer [22]. Most patients in our cohort died of cancer progression and cancer-related complications after esophagectomy, including pneumonia and sepsis, and these findings might support our speculation. The relationship between AF and pneumonia observed in our cohort, which is in line with the previous results [10,14], might as well reflect the underlying overwhelmed inflammation [18].

Furthermore, the impact of underlying inflammation on mortality would be more significant within the first year of esophagectomy, and this was reflected in our findings that a much higher incidence of 1-year mortality was observed in the AF group compared to the non-AF group and there was an association between AF and the 1-year mortality after excluding in-hospital mortality. Our results suggest that a close surveillance might be necessary in esophageal cancer patients who develop AF after esophagectomy during follow-up, especially within one year.

Despite adjustment for covariates in our model, our study was a retrospective observational study that had several inherent limitations to be considered. Confounders that were undetected in our dataset or have not been identified in previous studies were not adjusted in our statistical analyses. In contrast, there might be mediators in a causal pathway that were adjusted in the statistical analyses; it is difficult to discriminate mediators from confounders among the variables in our dataset. In addition, we could adjust only the data regarding the postoperative complications that were available in our dataset. Moreover, unfortunately there was no information regarding the accurate sequence of the postoperative complications and the causality between the postoperative AF and other complications. Therefore, caution has to be taken when interpreting the results. Despite the limitations of the study design, we think that our study is clinically significant as an exploratory research that suggests a perspective that postoperative AF might be an independent risk factor in esophageal cancer patients who underwent esophagectomy.

We also found that advanced age and preoperative CCB medication were associated with an increased risk for AF after esophagectomy. Consistent with the results of previous studies [23,24], advanced age was identified as a risk factor for AF after esophagectomy. It is well-established that aging leads to changes in the atrium, inducing atrial stiffening and splitting of the atrial excitation waveform [25,26]. The use of preoperative CCB medication also showed an unfavourable effect on the development of AF after esophagectomy in the present study. Dihydropyridine, a type of CCB that exclusively acts on the vasculature, results in vasodilation. The harmful effects of preoperative CCB medication on the development of AF observed in our present analysis might be partly attributable to the prevalent use of dihydropyridine-type CCBs, although we did not confirm this due to the lack of data in the postoperative period. Esophagectomy is usually a long surgery, and is often accompanied by unstable hemodynamics. Patients who receive preoperative dihydropyridine-type CCBs might be at a greater risk of developing hypotensive events. To resolve this promptly, fluid might be administered quickly, and thereafter, acute atrial dilation, which is a mechanism associated with the development of AF [27,28], might follow. Our results suggest that the CCB medication might be considered to be discontinued before esophagectomy, and if necessary, vasopressor should be considered to be administered to reverse hypotension during surgery in patients who had been taking CCB medication, especially for elderly patients.

In addition, the renin-angiotensin system and angiotensin II receptor are associated with structural and electrical remodelling of the atrium, which predisposes patients to AF [29–31]. Blockage of the renin-angiotensin system using ACEIs or ARBs has beneficial effects on the atrial remodeling in animal and human models [32–35]. The protective effect of the preoperative ACEI or ARB medication observed in our study might be in line with these previous results.

Our current study had the following limitations. First, the duration of the use of each medication was not considered in our analysis, and ACEI and ARB medications were not considered separately. Further studies that include specific data on the preoperative medications might be necessary. Second, we could not exclude gender as a risk factor for AF after esophagectomy despite the large number of studied patients, because the male patients accounted for approximately 94% of our entire cohort. Third, we did not include AF if the patient was asymptomatic at the general ward. Thus, there might be a possibility to have underestimated the incidence of AF. Further studies might be necessary to investigate whether AF with various hemodynamic status would similarly affect mortality.

In conclusion, newly developed AF during the postoperative period might be associated with mortality in esophageal cancer patients undergoing esophagectomy. This might suggest that the patients who developed AF after esophagectomy need a closer surveillance compared to the patients who did not during follow-up. Further studies will be needed to determine whether postoperative AF would have a causal relationship to mortality or would be a surrogate maker for sicker patients in those with esophageal cancer undergoing esophagectomy.

## Supporting Information

S1 Dataset This file is the dataset of our study. (XLSX)
